# Gonorrhoea in Greenland: geographic differences in diagnostic activity and incidence of gonorrhoea in 2015

**DOI:** 10.1080/22423982.2018.1445938

**Published:** 2018-03-04

**Authors:** Anne-Sophie Homøe, Sine Berntsen, Michael Lynge Pedersen

**Affiliations:** ^a^ University of Copenhagen, Copenhagen, Denmark; ^b^ Queen Ingrid Health Care Center, Nuuk, Greenland; ^c^ Greenland Center for Health Research, Institute of Nursing and Health Science, University of Greenland, Nuuk, Greenland

**Keywords:** Neisseria gonorrhoea, Incidence, diagnostic activity, venereal disease

## Abstract

For decades the spread of sexually transmitted infections (STIs) has been a health concern in Greenland, especially within the age group of 15–34 year olds. However, no overview exists of the potential differences in regional incidence and management of STIs. This study investigates the age, gender and region specific diagnostic activity and incidence of gonorrhoea in Greenland in 2015. The study design was an observational cross sectional register study with inclusion of patients tested for gonorrhoea in 2015. Patients above 15 years of age were included. Data was obtained from the laboratory system used at The Central Laboratory at Queen Ingrid’s Hospital in Nuuk. We found, in 2015, a total of *17,911* tests for gonorrhoea were performed on both men and women. Women accounted for *68*% of the tests, while men accounted for *32*%. The positivity rate was *7,878* pr. *100,000* of which *56*% were women and *44*% were men. The regional distribution showed a disparity of the testing rate and the rate of positive gonorrhoea tests.. Thus, we have documented a high diagnostic activity and high incidence of gonorrhoea in Greenland in 2015 among both women and men. We also found significant regional differences in both diagnostic activity and gonorrhoea incidence

## Introduction

Sexually transmitted infections (STIs) have been a health concern in Greenland for decades [1-7]. Epidemic peaks of STIs in Greenland were observed in the 1970s and 1980s with more than *12,000* cases of gonorrhoea in 1974 and around *13,000* cases in 1982 [8]. In the beginning of the 1990s a significant decrease in the incidence of STIs was seen, possibly as a result of the Stop-AIDS campaign and the efforts of venereologists and Greenlandic nurses educated in venereology [4,5]. However, throughout the recent decade the incidence of gonorrhoea in Greenland has begun to increase again (From *872* cases in 2002 to *1,550* cases in 2014 [8,9]). This increase in gonorrhoea incidence corresponds to an equivalent increase in both chlamydia and syphilis cases [10], and demonstrates the need for continuous focus on STIs in Greenland. Especially among young adults, an increased risk of infection has been detected [11].

In Greenland, free health care, as well as free contraceptives are provided for the entire population. Both nationally and locally the Greenlandic health authorities have launched several initiatives through the years in order to reduce the incidence of gonorrhoea and other STIs. Implementation of new diagnostic methods, enhancement of infection surveillance and screening, and public campaigns and interventions such as peer-to-peer programmes in schools are some of these initiatives [10,12,13]. Furthermore, sexual health education is mandatory in the public school in Greenland, all proving that authorities have an interest in improving sexual health in Greenland. However, currently, it seems that these initiatives are not enough to decrease the incidence of gonorrhoea [12].

Greenland reports the highest rates of infection with gonorrhoea in The North American Arctic [14]. The incidence of gonorrhoea is a matter of concern because of the possible severe sequelae after gonorrhoea infection such as salpingitis and pelvic inflammatory disease, which may lead to sterility and/or ectopic pregnancy.

Recently there has been focus on alternative strategies to deal with the increase in gonorrhoea and other STIs in Greenland. A more community-based approach, e.g. *Inuulluataarneq* (having the good life) involving family and youth in sexual health education programmes, storytelling and peer-to-peer sexual education are some of these strategies [4,12,13].

With a population of about 56,000 and an area of approximately 2 M km^2^, Greenland is the largest island in the world and also among the least densely populated. The population is widely spread along the coast in 18 towns and 60 settlements, making homogenous health care a continuous challenge. As a consequence there might be regional disparities in the local diagnostic activity and incidence of STIs throughout Greenland.

Currently, no evaluation on potential differences in regional incidence and management of STIs including prevention, screening procedures, diagnostic approaches and treatments in Greenland has been conducted.

Because of the challenges mentioned above, it seems crucial to know and understand the regional differences in treatment and diagnostics of gonorrhoea around Greenland in order to initiate strategies that are effective in every part of the island.

Hence, the objective of this study was to estimate age and gender specific diagnostic activity and incidence of gonorrhoea in different regions in Greenland in 2015.

## Methods

The study was designed as an observational cross sectional register study of all patients at or above 15 years of age tested for gonorrhoea in Greenland in 2015.

### Setting

Greenland is an autonomous part of the Kingdom of Denmark with the freedom to self-govern. The majority of the population is Inuit (*89%*) [15] and the remaining parts are mainly Danes. Nuuk with its *17,000* inhabitants is the largest town and the capital of Greenland. Greenland is further divided into 16 towns and adjacent settlement, all distributed into 5 regions. Each town has its own hospital or healthcare centre providing health care service to the adjacent settlements. All towns in Greenland are included in this study and the towns mentioned include the surrounding settlements and inhabited stations and sheep farms.

### Study population, routine testing methods and treatment

All patients were identified through a data extraction from the laboratory system used at The Central Laboratory at Queen Ingrid’s Hospital in Nuuk. Since 2011 the diagnosis of gonorrhoea in Greenland has been performed on urine samples with nucleic acid amplification tests (NAATs) by strand displacement amplification (Becton Dickinson ProbeTec). Urine samples are collected locally. Subsequently, the urine samples are sent to the central laboratory in Nuuk for analysis. All tests are marked with an area code and are analysed and registered at The Central Laboratory at Queen Ingrid’s Hospital in Nuuk. Thus, all patients diagnosed with gonorrhoea can be identified including age, gender and region where the tests have been obtained [10,16]. All patients with positive gonorrhoea tests are offered a combination treatment of 500 mg ceftriaxone i.m. and azithromycin 2 g according to European guidelines [10,17,18].

The indication for testing as well as the methods of contact tracing varies from region to region in Greenland. In Nuuk as well as in some of the other hospitals/healthcare centres, it is possible to get tested for gonorrhoea with minimal contact to healthcare personnel and without a physical examination. Furthermore, in these areas contract tracing is done by the patient him/herself (patient referral). This is in contrast to other hospitals/healthcare centres where it is routine to perform a medical examination prior to testing for gonorrhoea and where the contact tracing is done by healthcare professionals (provider referral).

### Ethics

The study complies with the Helsinki Declaration II and has been approved by The Ethics Committee for Medical Research in Greenland (no. KVUG-2016–16)) as well as the Agency for Health and Prevention in Greenland.

### Statistics

In order to maintain full anonymity of gonorrhoea cases, we chose to only include towns with 50 or more cases of gonorrhoea. In many of the towns the population is quite small, which prompted this lower limit. In our data set the largest towns in West Greenland (Nuuk, Sisimiut, Aasiaat, Ilulissat, Qaqortoq) and the largest town in East Greenland (Tasiilaq) were presented separately. The remaining population living in smaller towns and affiliated settlements was pooled into 1 large group to improve statistical analysis.

The age and gender specific proportions of the population tested for gonorrhoea and the incidence of gonorrhoea were calculated using the population in Greenland as per 1 July 2015 as a background population [15]. Diagnostic activity and incidence of gonorrhoea in the different regions of Greenland was compared for a selected age group (15–34 years old). Frequencies were compared using chi-square test (goodness of fit) [19]. Chi-square tests were performed on observed versus expected numbers between the tested groups. A p-value below 0.05 was considered significant.

## Results

Rates per *100,000* population of tests for gonorrhoea and positive tests for gonorrhoea, respectively, are shown in Table 1. Furthermore, the number (n) of tests and positive tests are disclosed. Rates and numbers (n) are stratified by gender and by age.

**Table 1.** Nationwide rates of gonorrhoea tests and positive gonorrhoea tests in Greenland in 2015.^a^

Age	Rate of all tests taken^b^ (n)				Rate of positive tests^c^ (n)				Positive tests out of all tests^d^		
Years	Total	Males	Females	p-Value^e^	Total	Males	Females	p-Value^f^	Total	Males	Females
Total	40,439	24,514	58,518	<0.001	3,524	2,904	4,228	**<0.001**	*8.7*	*11.8*	*7.2*
	(17,911)	(5,773)	(12,138)		(1,561)	(684)	(877)				
15–19	77,484	34,277	120,929	<0.001	9,016	4,719	13,336	**<0.001**	*11.6*	*13.8*	*11.0*
	(3,111)	(690)	(2,421)		(362)	(95)	(267)				
20–24	99,370	62,746	137,799	<0.001	10,829	9,960	11,740	0.056	*10.9*	*15.9*	*8.5*
	(4,423)	(1,430)	(2,993)		(482)	(227)	(255)				
25–29	83,157	53,720	113,275	<0.001	7,458	6,820	8,110	0.101	*9.0*	*12.5*	*7.2*
	(3,713)	(1,231)	(2,500)		(333)	(154)	(179)				
30–34	59,016	36,174	83,069	<0.001	3,949	3,874	4,028	0.801	*6.7*	*10.7*	*4.8*
	(2,376)	(747)	(1,629)		(159)	(80)	(79)				
35–39	44,216	28,208	63,108	<0.001	2,825	2,656	3,002	0.552	*6.4*	*9.4*	*4.8*
	(1,445)	(499)	(946)		(92)	(47)	(45)				
40–44	29,430	20,808	40,043	<0.001	1,261	1,230	1,298	0.867	*4.3*	*5.9*	*3.2*
	(910)	(355)	(555)		(39)	(21)	(18)				
45–49	17,649	12,533	23,521	<0.001	924	999	838	0.558	*5.2*	*8.0*	*3.6*
	(859)	(326)	(533)		(45)	(26)	(19)				
50–54	11,695	8,856	15,004	0.002	397	516	257	0.146	*3.4*	*5.8*	*1.7*
	(589)	(240)	(349)		(20)	(14)	(6)				
55–50	7,888	6,750	9,391	<0.001	432	491	354	0.516	*5.5*	*7.3*	*3.8*
	(310)	(151)	(159)		(17)	(11)	(6)				
60+	2,460	3,119	1,640	<0.001	168	230	92	0.159	*6.9*	*7.3*	*5.6*
	(175)	(122)	(53)		(12)	(9)	(3)				
15–34	80,338	47,359	114,397	<0.001	7,878	6,453	9,350	0.001	*9.8*	*13.6*	*8.2*
	(13,623)	(4,080)	(9,543)		(1,336)	(556)	(780)				

^a^Stratified by age and gender.

^b^Rate of all tests taken for gonorrhoea per 100,000.

^c^Rate of positive tests per 100,000.

^d^Percentage of positive tests out of all tests taken for gonorrhoea.

^e^p-Value in the columns for rate of all tests taken for gonorrhoea represents tests comparing the difference in gonorrhoea testing between males and females.

^f^p-Value in the rows for rate of positive tests represents tests comparing the difference in positive tests between males and females.

In 2015 *17,911* tests for gonorrhoea were performed in Greenland. Of these *12,138* tests (*68*%) were performed on women and *5,773* tests (*32*%) were performed on men.

### The number of tests was distributed in *9,854* individuals; *6,249 (63%)* women and *3,605 (37%)* men.

In total, *1,561* tests distributed on *1,365* individuals (*756* women, *609* men) were positive for gonorrhoea in 2015. Of these, *877* tests (*56*%) were positive in women and *684* tests (*44*%) were positive in men (Table 1).

### Age and gender

Women were tested more often than men in all age groups except for the oldest age group (60+ years). The highest number of tests was seen in women between 20 and 24 years of age (*2,993*). Of all tests, *76.1*% were performed in the group of 15–34 years of age (Table 1).

In this group, *1,336* positive tests were performed, corresponding to *85.5%* of all tests positive for gonorrhoea in Greenland in 2015.

In the group of women 15–34 years of age a total of *877* positive tests were reported, compared to only *684* positive tests for men in the same age group. However, in this age group, the rate of positive tests per total tests taken was significantly higher in men (*13.6*%) than in females (*8.2*%) (Table 1).

### Regional distribution

Table 2 provides an overview of rates per 100.000 population and number (n) of total tests for gonorrhoea and tests positive for gonorrhoea respectively, stratified by geographical region and gender. Only the group 15–34 years of age is included.

**Table 2.** Rates of gonorrhoea tests and positive gonorrhoea tests in Greenland in 2015 stratified by town.^a^

Town	Aasiaat	Ilulissat	Nuuk	Sisimiut	Tasiilaq	Qaqortoq	Rest of Greenland^b^	Total	p-Value^c^
Rate of all tests taken^d^									
Total	114,150	99,230	78,331	88,253	97,452	85,590	62,939	80,338	<0.001
(n)	(1,210)	(1,419)	(4,197)	(1,743)	(918)	(891)	(3,242)	(13,623)	
Females	164,285	134,281	113,499	120,682	139,549	115,458	90,752	114,397	<0.001
(n)	(874)	(991)	(3,052)	(1,132)	(681)	(605)	(2,208)	(9,543)	
Males	63,636	61,849	42,899	58,919	52,202	55,899	38,042	47,359	<0.001
(n)	(336)	(428)	(1145)	(611)	(237)	(289)	(1,034)	(4,080)	
Rate of positive tests^e^									
Total	10,471	15,314	5,814	9,569	7,112	11,527	6,154	7,878	<0.001
(n)	(111)	(219)	(313)	(189)	(67)	(120)	(317)	(1,336)	
Females	11,654	16,802	6,991	12,046	8,606	12,977	7,521	9,350	<0.001
(n)	(62)	(124)	(188)	(113)	(42)	(68)	(183)	(780)	
Males	9,280	13,728	4,638	7,328	5,506	10,058	4,930	6,453	<0.001
(n)	(49)	(95)	(125)	(76)	(25)	(52)	(134)	(559)	
Positive tests out of all tests^f^	*9.2*	*15.4*	*7.5*	*10.8*	*7.3*	*13.4*	*9.8*	*9.8*	

^a^Age 15–34 years.

^b^The remaining population living in smaller towns and affiliated settlements.

^c^p-Value in the rows for rate of all tests taken for gonorrhoea represents tests comparing the difference in gonorrhoea testing between the towns.

^d^Rate of all tests taken for gonorrhoea per 100,000.

^e^Rate of positive tests per 100,000.

^f^Percentage of positive tests out of all tests taken for gonorrhoea.

The highest number of tests for gonorrhoea was observed in Aasiaat with *164,285* tests per *100,000* for women and *63,636* per *100,000* for men. In Nuuk, the testing rate was somewhat lower (*78,331* per *100,000*).

The highest number of positive tests for gonorrhoea was observed in Ilulissat. For both men and women the rate was significantly higher than in the other regions, with *16,802* and *13,728* positive cases pr. *100,000*, respectively. The number of positive gonorrhoea tests was significantly lower in Nuuk and Tasiilaq for both men (*125* and *25*) and women (*188* and *42*) compared with other geographical regions in Greenland.

## Discussion

Our study presents an age, gender and region specific overview of gonorrhoea testing frequency and number of positive gonorrhoea tests in Greenland in 2015.

As shown in previous studies, we found that the number of gonorrhoea cases in Greenland is high. In Denmark, *2,788* cases of gonorrhoea (for all age groups) were reported in 2015 corresponding to approximately *49* cases per *100,000* [20]. This number is many times lower than the approximately *2,800* cases per *100,000* found in Greenland (for all age groups) the same year [9,21]. As previously mentioned, Greenland also has a higher number of gonorrhoea infections than the rest of the North American Arctic [14] where the living conditions are comparable to those in Greenland.

Our finding suggests that there are regional differences in Greenland in testing activity and rate of positive tests by tests taken.

The highest testing frequency was seen in Aasiaat, where the testing frequency was 1.5 times higher than in Nuuk.

The highest number of tests positive for gonorrhoea was found in Ilulissat and Qaqortoq. In Ilulissat the number of tests positive for gonorrhoea was 2.6 times higher than in Nuuk. It is surprising that Nuuk had the lowest reported number of positive tests and also one of the lowest testing frequencies (Table 2). There are many young people living in Nuuk and easy access to STI testing with minimal contact to healthcare personnel. Thus, a higher testing frequency and subsequent a higher number of positive tests was expected. Nuuk also had one of the lowest rates of positive tests by tests taken (*7.5%*). In Tasiilaq, the lowest rate of positive tests by tests taken was found (*7.3%*).

Of the *17,911* tests, *76*% were performed on 15–34 year olds. This is most likely due to higher sexual activity and more frequent change of sexual partners among the younger generation [11,22]. When looking at rates of positive tests by tests taken, it is clear that this number is significantly higher in the male group than in the group of women, *13.6%* and *8.2%*, respectively. This corresponds well with the higher frequency of tests in the group of women, which could be explained by routine prenatal testing and testing prior to abortion.

The high number of tests may be due to lack of treatment compliance. However, to investigate this is out of the scope of this study, thus we did not explore this further.

It can be speculated whether the regional variability in positive gonorrhoea tests seen in this study could in part be explained by differences in partner notification (patient referral vs. provider referral). However, recent studies have not been able to identify any single optimal strategy for partner notification for any particular STI [23].

Other hypotheses could be differences in living conditions as well as socio-economic and educational differences. There might be a difference in sexual behaviour among young people with different educational levels. The more highly educated populations are mostly situated in Nuuk.

In this study we have not included background characteristics of the people tested, but have solely looked at the tests performed. Thus, we are not able to say if or how these factors influence the rate of gonorrhoea in the different regions.

Since 2009 the number of gonorrhoea cases has exceeded *1,000* per year [9,18]. We have observed an overall increase in gonorrhoea cases in 2015, however there may be even more cases of gonorrhoea than reported, since the infection can be asymptomatic in women (*50*%) and in cases of urethral infections asymptomatic in men (*10*%) [24]. The group of asymptomatic cases make an important reservoir of infection and this constitutes a risk of continuous infection. The current increase could be due to lack of effective STI prevention strategies and/or an increase in diagnostic activity throughout all regions of Greenland as well as the implementation of NAAT-testing in 2011 [12,13,16] .

Many programmes have only been implemented locally and not nationally due to geographical challenges. This has made it difficult to maintain healthcare programmes, because of frequently changing healthcare personnel and programmes that are never deeply rooted into the healthcare system as part of a long-term solution [4,12,13].

Development of Neisseria gonorrhoea (GC) strains resistant to first-line treatment has evolved continuously in Greenland. This growth of antimicrobial resistance calls for an immediate effective approach in order to prevent and control the spread of gonorrhoea as well as other STIs [4,10,17,26,27].

### Strengths and limitations

The main strength of our study is that due to national surveillance and full central laboratory data we have the complete number of gonorrhoea tests and positive gonorrhoea tests nationwide in Greenland in 2015. Furthermore, the thorough registration of gonorrhoea tests have ensured a valid recording of the area/town were the various tests have been performed.

A limitation of this study is that we have focused on number of tests and not on number of persons. Therefore double registration cannot be excluded. However, we found that *1,561* tests were distributed on *1,365* individuals and therefore double registration is believed to be limited. We have not in this study investigated or described the specific distinctions in contact tracing and treatment approaches in the different areas/towns. This is a limitation to the study, as it is highly relevant when looking at regional differences. Our results indicate that this would be interesting to examine further in future studies.

Our study is a simple outline of age, gender and region specific differences in diagnostic activity concerning gonorrhoea. Yet, the limitations to this study raise some important questions for future studies to enable a useful map of the current gonorrhoea epidemic.

## Conclusion

The study shows that in 2015 there was a high diagnostic activity and a high incidence of gonorrhoea in Greenland. It also shows that there were disparities by age, sex and region in rates of tests for gonorrhoea and positive gonorrhoea tests in Greenland in 2015.

To our knowledge this is the first study examining regional differences in testing frequency and incidence of gonorrhoea in the different areas of Greenland.

Future studies could focus on the development of testing frequency and incidence of gonorrhoea regionally over the years. This would make it possible to detect if certain regions/towns have had an especially high increase or decrease in incidence over the last years. In continuation of this, as earlier mentioned, further exploration of regional differences in diagnostics, treatment and contact tracing would be highly relevant.

Our study indicates a need for an evaluation of the current national strategy. A more uniform nationwide approach to gonorrhoea prevention, diagnostics and treatment is perhaps required to prevent further spread.
